# Impediments to child education, health and development during the COVID-19 pandemic in India

**DOI:** 10.1016/j.lansea.2022.04.001

**Published:** 2022-04-19

**Authors:** Akshay Raut, Nguyen Tien Huy

**Affiliations:** a St. George's Hospital, Grant Government Medical College and Sir J.J. Group of Hospitals, Mumbai, India; b School of Tropical Medicine and Global Health (TMGH), Nagasaki University, Nagasaki 852-8523, Japan

As India nears normalcy after facing three waves of the COVID-19 pandemic, clearer evidence has emerged about the profound negative impact of the pandemic upon the well-being of children in the country. Although the interventions by the Union and the state governments have helped in minimising the disruptions to the overall child health and development, certain challenges still continue to prevail and need to be addressed.^1^^,^^2^

The Anganwadi Services Scheme, one of the largest and unique programmes globally for early childhood care and development, was greatly impacted due to the lockdown imposed by the government, which resulted in closure of Anganwadi centres across the country. This has affected the early childhood care and non-formal pre-school education services provided at such centres; which catered to around 24 million children between the ages of 3 to 6 years in 2020 before the lockdown.^1^ Furthermore, these centres also provided training to parents and caretakers about early stimulation activities for psychosocial development of children from birth up to 3 years of age which has also been affected due to the lockdown. Such disruptions in early childhood stimulation and pre-school education can have long-term negative consequences on the physical and mental health of children.^3^^,^^4^ The authorities need to urgently undertake steps to assess the situation and introduce comprehensive remedial services to catch-up with the losses in early childhood education at all levels; ensure prompt coordination with the local workforce to increase parent and community engagement and ensure restoration of these services at the earliest.

About 264.5 million children have been affected throughout the country as a result of closure of schools and consequent disruption of the formal school education system in 2020-2021.^2^ As per the enrolment and population projection data obtained from the Unified District Information System for Education Plus (UDISE+) report which is the online management information system for school education in India; around 108 million children were out-of-school from pre-primary to higher secondary section as a result of non-enrolment alone during 2020-2021.^5^ A decrease in enrolment of 2.9 million children in pre-primary section and 1.8 million children in class 1 of the primary section was observed in 2020-2021 as compared to the previous year.^5^ Additionally, a decrease of 3.55% in the total enrolment of children with special needs was evident in 2020-2021.^5^ On the contrary, a decrease in dropout rate was found during 2020-2021 as compared to the previous year.^5^ Even though the enrolment and dropout statistics were not drastically affected during the pandemic, mere enrolment and absence of dropouts did not necessarily infer that the children attended school during the pandemic.^6^ The impact was further augmented by the resultant economic disparities, food shortages and reverse migration occurring across the country which contributed to further distancing of children from education.^6^ Additionally, a disproportionate rise in the number of out-of-school girls is expected because of widening of the prevailing socio-economic disparities in education of girls.^6^^,^^7^ The gendered impact of the pandemic needs to be addressed and measures need to be undertaken which are inclusive and supportive for such vulnerable populations.^7^ Furthermore, the government needs to promptly identify such out-of-school children and enrol them into the formal school education system. Upon conducting two door-to-door survey-cum-enrolment drives in 2020-2021, the state of Bihar alone identified and enrolled a total of 5.2 million out-of-school children.^2^ Other states and union territories need to undertake similar comprehensive strategies to decipher the true estimate of out-of-school children.

Even though virtual learning has helped in minimising the losses to education during closure of schools, there has been an increased risk of missing out on education for children with lack of access to digital devices. About 29.6 million children in India have been found to have no access to a digital device through surveys conducted in multiple states and union territories as of June 30, 2021.^1^ Furthermore, studies conducted in multiple states across India have also identified a gender gap in access and use of digital devices for learning; with girls having lesser access to devices and a higher comparative time utilisation in domestic chores and caregiving responsibilities rather than in learning.^6^^,^^8^ Economic constraints were the most common reason noted for lack of a digital device and access to the internet at home.^6^^,^^8^ Even the presence of a digital device with internet access did not ensure adequate children's access to virtual learning.^6^, ^7^, ^8^, ^9^, ^10^ A recently conducted study in January 2021 in 16,067 children in public schools across 5 states showed that about 92% and 82% children on an average had lost at least one specific language and one specific mathematical ability, respectively, as compared to previous year across all the primary classes.^11^ The most common lost specific language and mathematical abilities varied across the primary classes with reading fluency, basic mathematical operations and problem-solving skills affected in children belonging to classes 2 and 3; oral expression, place value and measurement skills were affected in classes 4 and 5 children, while writing and measurement skills were affected in class 6 children.^11^ Another series of surveys conducted in 2021 among 54,000 school students in three states showed poorer text reading levels and arithmetic skills consisting of recognising numbers and basic mathematical operations in children as compared to previous years indicating a clear loss of learning.^12^, ^13^, ^14^ The advent of virtual education during the pandemic has also resulted in further understanding of the existent social divide resulting from inequitable digital access across the states and amongst the various socio-economic strata and vulnerable groups in the country.^6^, ^7^, ^8^, ^9^, ^10^ The loss of learning due to digital divide further exacerbates the existing inequalities with severe consequences on the well-being of children and necessary measures must be taken to reduce the learning crisis in the country.^15^

Another aspect of the use of digital devices by children for educational purposes during school closures is the subsequent increase in screen time. Additionally, a decrease in duration of outdoor activities due to the lockdown and other pandemic-related restrictions has further augmented the use of digital devices in children. Even though the guidelines provided by the Indian Academy of Pediatrics suggests no screen exposure for children below 2 years of age, a supervised screen time of 1 hour and 2 h per day, respectively, for children of ages 2-5 and 5-10 years with a balance between screen time and outdoor activities, sleep, and interpersonal interactions; the pandemic has greatly affected the overall screen time in children.^16^ A study conducted during October to December 2020 in 1237 children of ages 9 to 14 showed a decrease in duration of outdoor activities from 8.5 h per week before the lockdown to 1.6 h per week after the lockdown and a 69% increase in screen time from 6.2 h per week before the lockdown to 19.8 h per week after the lockdown period.^17^ The study also found that increase in screen time on electronic gadgets was greater in boys and in children from private schools than government schools while the increase in screen time on television was more in girls; a pattern similar to the pre-pandemic period.^17^ An increased screen time in children was found to be associated with obesity, sleep, postural and visual disturbances, and cognitive disturbances amongst others.^16^ A few studies in school children have also identified the burden of increased screen time during the pandemic resulting in digital eye strain and other ocular problems along with postural and sleep disturbances in school children.^18^^,^^19^ There is a need to increase awareness about the ill-effects of increased screen time on the health of children alongside educating children and caregivers on health-promoting activities.

Although India has made progress in tackling the issue of child nutrition-related problems in the last few decades, the existing deficiencies in child nutritional status indicators have been further amplified due to the pandemic. Even though the Prime Minister's Overarching Scheme for Holistic Nourishment Programme (POSHAN Abhiyan) continues to tackle the problem of malnutrition in India by providing supplementary nutrition to 49 million children less than 6 years of age; the prodigious problem still persists.^20^^,^^21^ A total of 1.2 million moderately and severely malnourished children were identified in the country by the POSHAN Abhiyan till the end of April 2020 which rose to approximately 3.3 million children as of October 14, 2021 as estimated by the POSHAN Tracker application under the POSHAN Abhiyan.^20^^,^^22^ These numbers could be an underestimate as it only takes in account the children who are currently enrolled in the programme with available anthropometric records. While the problem of undernutrition remains the most common childhood nutritional-related disorder in the country, the problem of overnutrition and obesity is at-risk of increasing at a faster pace because of lifestyle modifications during the pandemic such as physical inactivity, increased screen time, unhealthy diet and disturbed sleep patterns.^23^^,^^24^ A study conducted in school children aged 10-15 years of age reported an increased consumption of junk food during the pandemic with a simultaneous decrease in physical activity.^23^ Another study conducted in 1692 school children belonging to the age group of 3–15 years showed an increase in body mass indices in all the participants with a 4.8% and 2.4% increase in the prevalence of overweight and obesity respectively in the participants one year after the announcement of lockdown.^24^ Both undernutrition and overnutrition have long-term negative consequences on child health and development and there is a need for augmenting the existing nutritional programs, ensuring food security and economic support, increase of family and community engagement in nutritional awareness and education programs.

The pandemic-related disruptions to the childhood vaccine delivery system have resulted in an additional rise of 1.4 million incompletely vaccinated children in 2020 as compared to 2019.^25^ The authorities need to further strengthen the existing policies and programmes alongside supply of essential health and immunization services, and ensure that such high impact interventions make appropriate modifications to reach every household in the country.^25^

The pandemic-related restrictions and disruptions in the access and utilisation of health services across the country has affected diagnosis of chronic illnesses in children. According to the data obtained from the Health Management Information System under the Union Health Ministry, there has been a significant decrease of 32% in children reported with tuberculosis during the period of April 2020 to March 2021 as compared to the previous year.^26^ The diversion of healthcare resources towards management of COVID-19 has affected the routine healthcare services. The reduced detection and delayed treatment of diseases like tuberculosis coupled with decrease in routine immunization programs can promote further spread of the communicable disease and contribute to further burden on the healthcare system. The Rashtriya Bal Swasthya Karyakram (RBSK), an ambitious government initiative for early child health screening and intervention services was severely affected during the pandemic period; the number of children screened between 6 months and 18 years decreased from 186 million to 22 million with a 93% decrease in children identified with nutritional deficiency and a 85% decrease in children identified with developmental delay during April 2020 to March 2021 as compared to the previous year.^26^ There is an urgent need to restore these services at the earliest to prevent long-term consequences on the health and development of children across the country. Similarly, management of chronic illnesses in children has also been affected indirectly due to the pandemic. The glycaemic control in children with Type 1 diabetes mellitus was found to be worsened during the period of lockdown due to unavailability of insulin and glucose testing strips as a result of limited stock, financial constraints, altered eating habits and decreased physical activity.^27^ A recent multicentric study conducted across the country concluded that every 2 out of 3 children with cancer had either a delayed diagnosis or treatment during the lockdown period.^28^ The pandemic and the resulting restrictive measures have also affected children with special needs and their caregivers across the country.^29^^,^^30^ In a survey conducted in caregivers of children with cerebral palsy, it was found that the pandemic affected routine care which resulted in worsening of physical functions and existing deformities along with changes in behavioral patterns of the children.^30^ A similar disruption of routine care with increased behavioral problems was observed in children with attention-deficit hyperactivity disorder and autism spectrum disorders.^31^^,^^32^ The pandemic-related restrictions have certainly affected the management of chronic childhood illnesses which could potentially increase the morbidity and mortality from these illnesses during the pandemic period rather than COVID-19 itself. There is a need to address these issues at the earliest and ensure that such children are supported and are at focus while instituting restrictive measures in future to prevent further harm to children suffering from chronic illnesses.

A disproportionate impact of the pandemic has been placed on the vulnerable and the marginalized children in India. A recent survey conducted across multiple states in marginalized and migrant children concluded that apart from the economic constraints, food shortages and obstacles to learning; some children also reported violence in their homes, experienced increase in negative feelings, reported stress or violence in household relationships, emotional distress due to lesser or no contact with friends and an increased involvement in household chores.^33^ A noticeable 50% rise in the number of calls for aid and assistance to CHILDLINE 1098, a free emergency phone service for children, was observed during the period of lockdown in March-April 2020.^34^ Amongst 0.46 million calls made between March 20 and April 10, 2020 which resulted in 9,385 direct interventions by the relevant organizations; about 20% of the interventions responded to child protection issues such as preventing child marriage, child abuse, trafficking, abandonment, neglect, and child labor. A total of 3,653 interventions responded to rescue of child laborers with nearly half of the rescued children between the ages of 11 to 15 years during the months of April to May 2020.^35^ Around 898 child marriages have been prevented since the lockdown.^34^ One of the grave impacts of the pandemic has been imposed upon children who have been orphaned due to loss of one or both parents or caregivers, and the children who have been abandoned during the pandemic period. As per the data obtained from the Union Ministry as on February 15, 2022, about 0.15 million children in India have lost either or both parents or have been abandoned due to any reason since April 1, 2020.^36^ Such events can have both short as well term long-term effects on emotional and behavioral health of children.^37^ There is a need for prompt interventions and policy measures to address and support the needs of children belonging to the vulnerable and marginalized population in the country.

The COVID-19 pandemic has also significantly affected the mental health of children globally. A recent meta-analysis comprising nearly 23,000 children, adolescents and caregivers globally found that nearly 4 out of 5 children had a negative psychological effect due to the pandemic and its consequent restrictions.^38^ The most predominant new-onset psychological symptoms observed in children were anxiety, depression, irritability and inattention.^38^ A striking increase in the number of reported deaths due to suicide in children below 18 years of age has been observed in the country in 2020. As compared to 2019, there has been an increase of 18.5% in the total number of deaths due to suicide in children with a 49% increase in the number of deaths due to suicide due to mental illness.^39^ These findings continue to further elucidate the need for robust psychosocial support programmes with prime focus on the mental health problems in children.

The COVID-19 pandemic has become a global health crisis with implications on all sections of the society, with children being the unheard victims of this pandemic. The indirect impacts of the pandemic on children in India have been widespread with inequity on socio-economic grounds along with wider ramifications on children belonging to the vulnerable and marginalized population. The extent of the problems faced by children during the pandemic need to be further identified, monitored and addressed. Additionally, further research needs to be conducted in future to draw conclusions on the long-term impact of the pandemic on the well-being of children. An urgent need for action is warranted at all levels of the society in order to prevent the profound negative effects on child education, health and development across the country thereby neutralising the progress made in the field across decades.

## Contributors

Akshay Raut – conceptualization, investigation, visualization, writing-original draft, writing-review and editing. Nguyen Tien Huy – supervision, writing-original draft, writing-review and editing

## Declaration of interests

We declare no competing interests.
